# Telangiectasia-Related Social Media Posts: Cross-sectional Analysis of TikTok and Instagram

**DOI:** 10.2196/41716

**Published:** 2023-02-16

**Authors:** Carrie Diamond, Alyssa P Quinn, Colby L Presley, Jennifer Jacobs, Melissa R Laughter, Jaclyn Anderson, Chandler Rundle

**Affiliations:** 1 Duke University School of Medicine Durham, NC United States; 2 Rocky Vista University College of Osteopathic Medicine Ivins, UT United States; 3 Department of Dermatology Lehigh Valley Health Network Allentown, PA United States; 4 Department of Dermatology New York University New York City, NY United States; 5 Department of Pathology Stanford University Stanford, CA United States; 6 Department of Dermatology Duke University Health System Durham, NC United States

**Keywords:** social media, telangiectasias, varicose veins, health information, misinformation, dermatology, health education, dermatologic information, health content, accuracy, educational content

## Introduction

While social media is increasingly used by dermatologists to educate the public [1], any social media user can freely create and disseminate content. As a result, the public may interact with errant recommendations or dermatologic misinformation [1]. Myths surrounding telangiectasias, a condition affecting up to 79% of men and 88% of women, encapsulate this problem [2]. We analyzed published content and its authors on TikTok and Instagram to appraise telangiectasia-related content.

## Methods

TikTok and Instagram were selected for their size and dearth of published literature, compared to the known presence of misinformation on platforms like Twitter and Facebook [1]. On TikTok and Instagram, #spiderveins was searched. The top 13 hashtags were collected from captions of posts including #spiderveins. The 10 most popular posts were analyzed for each hashtag. Non-English posts were excluded. Posts without medical explanations or marketing intent were excluded. The remaining posts were classified as educational, promotional, or advertisement, as in prior research studies [3]. Educational posts explained dermatologic conditions or procedures, promotional posts endorsed a practice or provider without offers for purchase, and advertisements offered services or products for purchase. Creator classification, post type, and post engagement were also collected.

## Results

Nondermatologists made up the majority (50/74, 68%) of telangiectasia-related content on TikTok (Table 1). Of 123 posts, 80.4% (n=99) of posts were educational, 11.4% (n=14) were advertisements, and 5.7% (n=7) were promotional; 57.7% (n=71) of posts were published by medical providers or practices, 16.3% (n=20) by influencers, and 26% (n=32) by businesses. On Instagram, #varicoseveins and #spiderveinremoval were the most popular hashtags for terminology and treatment, respectively (Table 2). From 117 posts, educational (n=59, 50.4%) content was once again the most common, followed by inspirational (n=27, 23.1%) and promotional (n=31, 26.5%). Influencers were responsible for 16.2% (n=19) of posts, medical providers for 59% (n=69), and businesses for 24.8% (n=29).

**Table 1:** Varicose veins and telangiectasia search terms, average user engagement, post content, type, and creator on TikTok.

Hashtag	Average likes	Average comments	Average shares	Content: advertisement, n (%)	Content: educational, n (%)	Content: promotional, n (%)	Post type: video, n (%)	Post source: influencer, n (%)	Post source: medical provider, n (%)	Post source: business, n (%)
#spiderveins	4152	143.7	639.8	1 (10)	8 (80)	1 (10)	10 (100)	3 (30)	4 (40)	3 (30)
#varicoseveins	32,493.80	214.9	11,171.80	0 (0)	9 (90)	1 (10)	10 (100)	3 (30)	7 (70)	0 (0)
#varicose	2551.10	56.5	379.6	0 (0)	9 (90)	1 (10)	10 (100)	4 (40)	6 (60)	0 (0)
#legveins	2050	40.8	111.1	5 (50)	4 (40)	1 (10)	10 (100)	0 (0)	6 (60)	4 (40)
#varicosevein	1244.10	42.9	523.1	0 (0)	10 (100)	0 (0)	10 (100)	2 (20)	8 (80)	0 (0)
#spidervein	89.5	6.4	2.8	0 (0)	10 (100)	0 (0)	10 (100)	3 (30)	4 (40)	3 (30)
#telangiectasia	41.7	0.333	1	0 (0)	10 (100)	0 (0)	10 (100)	0 (0)	10 (100)	0 (0)
#sclerotherapy	399,510.10	440.1	13,684.30	0 (0)	10 (100)	0 (0)	10 (100)	2 (20)	8 (80)	0 (0)
#varicosetreatment	1493.80	27.6	291.3	0 (0)	10 (100)	0 (0)	10 (100)	1 (10)	8 (80)	1 (10)
#veintreatment	1136.70	18.3	38	3 (30)	5 (50)	2 (20)	10 (100)	0 (0)	5 (50)	5 (50)
#spiderveinremoval	308.5	11.9	15.7	2 (20)	8 (80)	0 (0)	10 (100)	1 (10)	4 (40)	5 (50)
#laserveinremoval	399.5	7.4	13.1	5 (50)	5 (50)	0 (0)	10 (100)	0 (0)	2 (20)	8 (80)
#spiderveintreatment	284.8	6.5	5.9	1 (10)	8 (80)	1 (10)	10 (100)	1 (10)	5 (50)	4 (40)

**Table 2:** Varicose veins and telangiectasia search terms, average user engagement, post content, type, and creator on Instagram.

Hashtag	Average likes	Average comments	Post type: photo	Content: inspirational	Content: educational	Content: promotional	Post type: video	Post source: influencer	Post source: medical provider	Post source: business
#spiderveins	221.125	31.111	8 (89)	6 (67)	2 (22)	1 (11)	1 (11)	7 (78)	1 (11)	1 (11)
#varicoseveins	617.75	25.778	8 (89)	4 (44)	4 (44)	1 (11)	1 (11)	5 (56)	3 (33)	1 (11)
#varicose	534.25	55	9 (100)	2 (22)	5 (56)	2 (22)	0 (0)	2 (22)	7 (78)	0 (0)
#legveins	268.333	7.556	6 (67)	3 (33)	4 (44)	2 (22)	3 (33)	1 (11)	6 (67)	2 (22)
#varicosevein	184.667	12.667	2 (22)	0 (0)	5 (56)	4 (44)	7 (78)	0 (0)	7 (78)	2 (22)
#spidervein	250	34.889	8 (89)	0 (0)	8 (89)	1 (11)	1 (11)	0 (0)	8 (89)	1 (11)
#telangiectasia	406.833	16.556	6 (67)	2 (22)	6 (67)	1 (11)	3 (33)	0 (0)	4 (44)	5 (56)
#sclerotherapy	198	24.111	3 (33)	3 (33)	5 (56)	1 (11)	6 (67)	0 (0)	8 (89)	1 (11)
#varicosetreatment	89.714	5.333	7 (78)	3 (33)	5 (56)	1 (11)	2 (22)	3 (33)	5 (56)	1 (11)
#veintreatment	193.571	16.556	7 (78)	1 (11)	4 (44)	4 (44)	2 (22)	0 (0)	6 (67)	3 (33)
#spiderveinremoval	565	22.222	5 (56)	1 (11)	4 (44)	4 (44)	4 (44)	1 (11)	6 (67)	2 (22)
#laserveinremoval	146.8	108.889	5 (56)	0 (0)	4 (44)	5 (56)	4 (44)	0 (0)	3 (33)	6 (67)
#spiderveintreatment	93	14.333	2 (22)	4 (44)	4 (44)	1 (11)	7 (78)	1 (11)	5 (56)	3 (33)

## Discussion

Many patients use social media platforms for dermatologic information [4]. Our findings demonstrate the potential for the dissemination of misinformation from nonmedical users, with 35.7% (39/108) and 46.6% (61/132) of content from disease nomenclature and treatment generated by influencers and businesses, respectively. Prior research has demonstrated that information not produced by board-certified dermatologists has a higher propensity to be inaccurate [5]. There are existing features within social media platforms, like the duet feature on TikTok, where two videos are played simultaneously, that medical providers can use to their advantage to combat misinformation [3]. This study serves as a reminder that dermatologists should warn patients about inaccurate dermatologic information potentially found on social media apps and can do so in simple targeted messages. The limitations of our study include the evaluation of only two social media platforms, with a predominantly English userbase.

This study provides a sample of content creators in telangiectatic-related content. This study reinforces the importance of social media presence of board-certified dermatologists to comment on and combat inaccuracies by creating educational content and reacting to erroneous information. Further research is necessary to evaluate the scope of misinformation and its deleterious effects.
